# Loss of synaptic Zn^2+^ transporter function increases risk of febrile seizures

**DOI:** 10.1038/srep17816

**Published:** 2015-12-09

**Authors:** Michael S. Hildebrand, A. Marie Phillips, Saul A. Mullen, Paul A. Adlard, Katia Hardies, John A. Damiano, Verena Wimmer, Susannah T. Bellows, Jacinta M. McMahon, Rosemary Burgess, Rik Hendrickx, Sarah Weckhuysen, Arvid Suls, Peter De Jonghe, Ingrid E. Scheffer, Steven Petrou, Samuel F. Berkovic, Christopher A. Reid

**Affiliations:** 1 Epilepsy Research Centre, Department of Medicine, University of Melbourne, Austin Health, Heidelberg 3084, Victoria, Australia; 2 The Florey Institute for Neuroscience and Mental Health, The University of Melbourne, Parkville 3052, Victoria, Australia; 3 School of Biosciences, University of Melbourne, Parkville 3052, Australia; 4 Neurogenetics Group, Department of Molecular Genetics, VIB, Belgium; 5 Laboratory of Neurogenetics, Institute Born-Bunge, University of Antwerp, Belgium; 6 Department of Paediatrics, University of Melbourne, Royal Children’s Hospital, Parkville 3052, Victoria, Australia; 7 Division of Neurology, Antwerp University Hospital, Antwerp, Belgium

## Abstract

Febrile seizures (FS) are the most common seizure syndrome and are potentially a prelude to more severe epilepsy. Although zinc (Zn^2+^) metabolism has previously been implicated in FS, whether or not variation in proteins essential for Zn^2+^ homeostasis contributes to susceptibility is unknown. Synaptic Zn^2+^ is co-released with glutamate and modulates neuronal excitability. *SLC30A3* encodes the zinc transporter 3 (ZNT3), which is primarily responsible for moving Zn^2+^ into synaptic vesicles. Here we sequenced *SLC30A3* and discovered a rare variant (c.892C > T; p.R298C) enriched in FS populations but absent in population-matched controls. Functional analysis revealed a significant loss-of-function of the mutated protein resulting from a trafficking deficit. Furthermore, mice null for ZnT3 were more sensitive than wild-type to hyperthermia-induced seizures that model FS. Together our data suggest that reduced synaptic Zn^2+^ increases the risk of FS and more broadly support the idea that impaired synaptic Zn^2+^ homeostasis can contribute to neuronal hyperexcitability.

Febrile seizures (FS) are the most common seizure syndrome, affecting 2–3% of children in the pre-school years^1^. FS account for over 1 in 200 paediatric emergency department (ED) admissions manifesting in physical, psychological, and behavioural issues^2^. They may also be associated with more severe forms of epilepsy in later life with long-term studies indicating that 7% of children with FS subsequently develop epilepsy^3^. Despite the clinical burden little progress in understanding the causes of FS has been made over the last decade, making this area a key research priority for the epilepsy field^4^.

Several studies support the idea that low zinc (Zn^2+^) levels increase seizure susceptibility. For example, altering dietary Zn^2+^ intake can alter seizure susceptibility in a genetic mouse model of epilepsy, with low Zn^2+^ increasing sensitivity and high Zn^2+^ being protective^5^. Furthermore, rats administered intraperitoneal injections of the Zn^2+^ chelator sodium diethyldithiocarbamate develop seizures^6^. Importantly, Zn^2+^ levels are significantly lower in blood and/or cerebrospinal fluid of children that suffer FS; both when compared to healthy controls and when compared to children either presenting with fever alone or seizures not associated with fever^7^,^8^,^9^,^10^. These studies highlight dysfunction of Zn^2+^ homeostasis as a potential mechanism of enhanced FS susceptibility.

Genetic factors play an important role in determining FS susceptibility^11^,^12^. However, whether or not genetic variation in proteins essential for Zn^2+^ homeostasis contributes to FS susceptibility is not known. Zn^2+^ transporter 3 (ZNT3), encoded by *SLC30A3,* is well placed to modulate neuronal excitability. ZNT3 is primarily responsible for the transport of Zn^2+^ into synaptic vesicles where it is co-localised with glutamate and released in an activity-dependent manner^13^,^14^. High Zn^2+^ concentrations can occur in the extracellular space potentially regulating pre- and post-synaptic membrane excitability by modulating a variety of ion channels, receptors and transporters^15^. Synaptic Zn^2+^ released during short trains of activity inhibits NMDA receptors and hence acts as an important inhibitor of hippocampal neuronal circuit excitability^14^. Consistent with this, ZnT3 knock-out mice display increased susceptibility to pharmacological pro-convulsants^16^. Thus reduction in synaptic Zn^2+^ may increase neuronal excitability and consequently seizure susceptibility. Based on the central role of synaptic Zn^2+^ in modulating hippocampal excitability and clinical evidence implicating low cerebrospinal fluid and blood levels in FS we hypothesised that variation in ZNT3 would contribute to FS susceptibility. To address this we took a candidate gene approach, screened *SLC30A3,* and functionally validated a variant enriched in FS patients.

## Results

### *ZNT3* sequencing reveals a R298C variant enriched in FS patients

**Figure 1:**
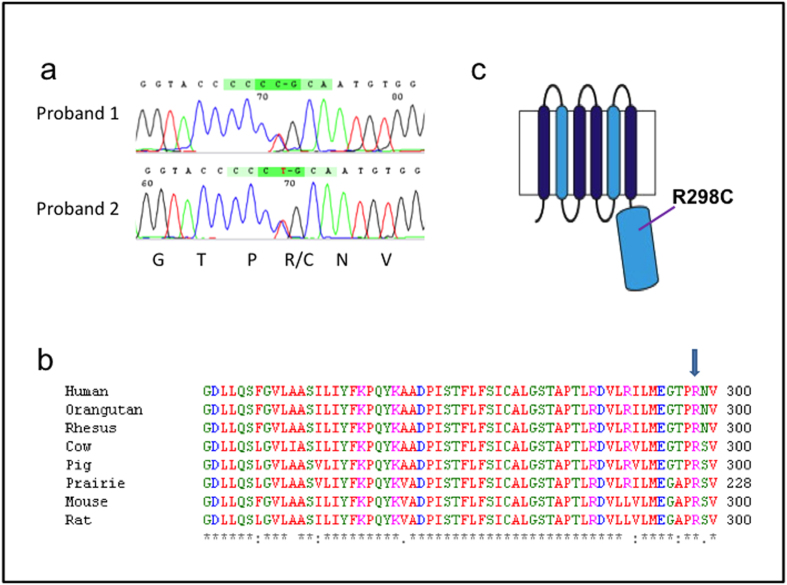
Enrichment of SLC30A3 variant in FS. (**a)** Sequence chromatogram showing the c.892C > T *SLC30A3* variant enriched in FS patients. (**b)** Multiple species alignment of ZNT3 protein sequence showing the R298 amino acid is highly conserved (arrow). Rhesus = Rhesus monkey; Prairie = Prairie vole. (**c)** Schematic showing domain structure of the ZNT3 protein. Light-blue shading indicates domains involved in Zn^2+^ binding. The R298C variant is located in the cytoplasmic domain near the C-terminus. Adapted from^40^.

Our screen of FS probands for variants in the coding and splice site regions of *SLC30A3*, encoding human ZNT3, revealed a variant (c.892C > T; p.R298C) in the cytoplasmic domain (Fig. 1a,c). This variant is enriched in FS probands (n = 3/286; 1%) and absent from population-matched controls (n = 0/643, P < 0.05, Fisher test). Two probands were from Australia (Probands 1 and 2) and one from Belgian (Proband 3). The variant is reported at over 10-fold lower frequency amongst the 6,491 exomes on the Exome Variant Server (0.09%; EVS; http://evs.gs.washington.edu/EVS/) and the 60,386 exomes on the Exome Aggregation Database (~0.08%; ExAC; http://exac.broadinstitute.org/).

The case-control analysis of this variant in FS probands against the EVS gives an odds ratio of 11 (CI 2–37). Given a lifetime prevalence of 2–3%, the 10 fold increase in risk implied by this odds ratio would lead to an absolute risk of over 1 in 5 of developing FS. High Grantham (180) and PolyPhen-2 (0.995/1) scores also suggest that the variant is probably damaging to the ZNT3 protein. Furthermore, the amino acid change occurs in a highly conserved region of the cytoplasmic domain (Fig. 1b,c) and *SLC30A3* has low tolerance to variation (intolerance score = −0.8; 12th percentile)^17^. The variant substitutes a positively charged, polar arginine residue at position 298 with a cysteine residue possessing a thiol side chain that is susceptible to oxidation and may form disulphide bonds with other cysteine residues.

### Inheritance of the R298C variant

**Figure 2:**
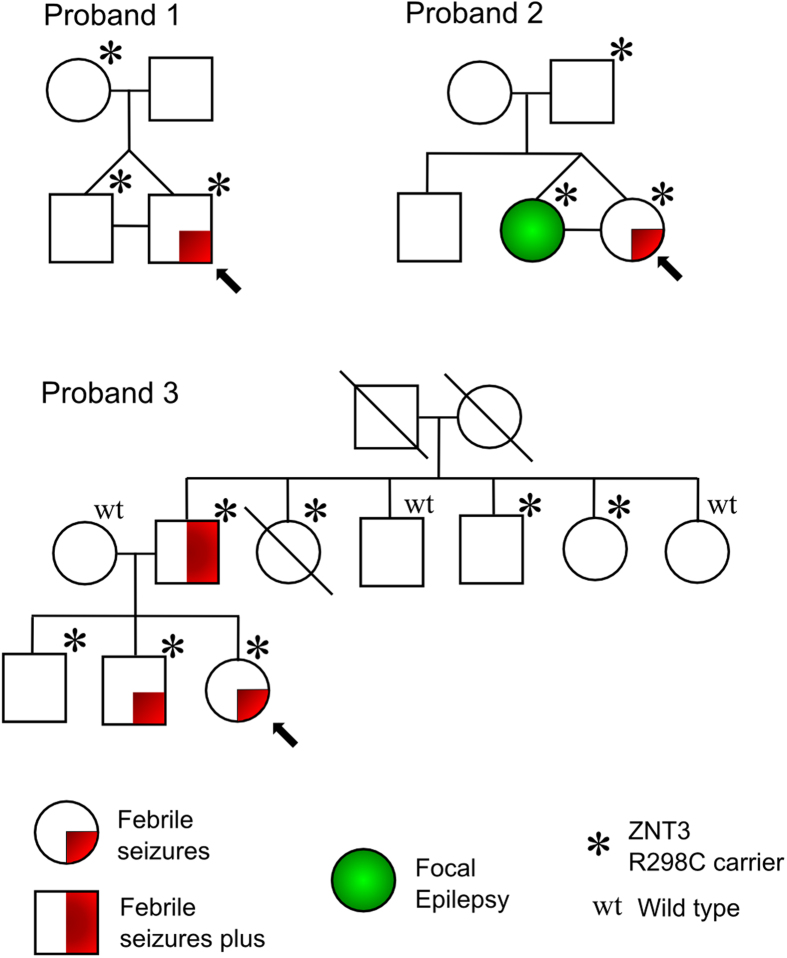
Pedigrees of probands carrying ZNT3 variant. All three probands carry the c.892C > T (p.R298C) variant in *SLC30A3*. The segregation of the variants in their respective pedigrees is shown: *heterozygous c.892C > T (p.R298C) genotype (C/T); wt, homozygous wild-type genotype (C/C).

All three probands had simple FS. A twin cohort made up a significant component of the sample (37/259) and probands 1 and 2 both have a monozygotic twin who has the R298C allele. The twin of proband 1 is unaffected while the twin of proband 2 had afebrile focal dyscognitive seizures in childhood (Fig. 2). Both inherited the variant from an unaffected parent. Proband 3 inherited the variant (Fig. 2) from her father, who was affected with febrile seizures intermixed with afebrile seizures until the age of 14 (febrile seizures plus, FS+). A brother was also affected with FS while there are another 4 unaffected carriers of the allele. Overall, the phenotypes in the relatives strengthen the argument that R298C is a risk allele rather than a Mendelian dominant allele, and suggest an association with epilepsies wider than simply FS. Marker analysis was performed on the three probands and available relatives using microsatellites at the *SLC30A3* locus (Supplementary Table 1). This revealed a small region of between 1.84 Kb and 1.50 Mb shared by all three probands (1, 2 and 3), and a larger region of between 1.03 Mb and 1.76 Mb shared by only the two Australian probands (1 and 2).

### ZNT3 (R298C) causes a trafficking deficit resulting in severe loss-of-function

**Figure 3:**
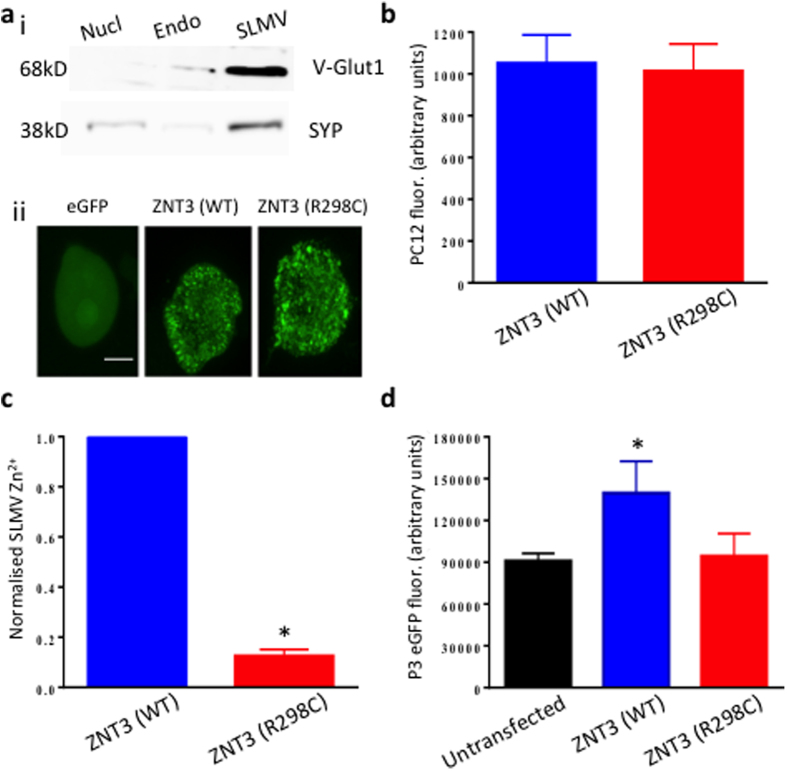
Functional analysis reveals a loss-of-function due to the ZNT3 (R298C) variant. ai. Western blot analysis of the three fractions isolated by differential centrifugation including: cell/nuclear (Nucl.), endosomal/mitochondrial (Endo) and synaptic –like microvesicle (SLMV) fractions. Cross-reactivity with synaptophysin (SYP) and vGlut1 antibodies is greatest in the P3 (200,000 g) fraction confirming SLMV enrichment. **aii** Confocal images of representative PC12 cells transfected with eGFP, ZNT3 (WT)-eGFP and ZNT3 (R298C)-eGFP. Scale bar = 15 μm **(b**) Median eGFP fluorescence in PC12 cells transfected with ZNT3 (WT) and ZNT3 (R298C) confirming similar ZnT3 translation from both constructs (p = 0.74, n = 4, Student’s paired t-test).(**c**) Normalized Zn^2+^ concentration in the SLMV fraction of ZNT3 (WT) and ZNT3 (R298C) transfected PC12 cells from four independent experiments. Zn^2+^ was significantly reduced in ZNT3 (R298C) SLMVs (*p = 0.0013, n = 4, Student’s ratio paired t-test) (**d**) Mean fluorimetry measurements of eGFP in the SLMV fraction of ZNT3 (WT) and ZNT3 (R298C) transfected and in non-transfected PC12 cells. Fluorescence was significantly reduced in ZNT3 (R298C) relative to ZNT3 (WT) and was indistinguishable from auto-fluorescence in non-tranfected cells (*p = 0.0001, n = 3 for all groups, One way ANOVA).

We developed a functional assay based on an established method in which ZNT3 is transfected into rat pheochromocytoma (PC12) cells^18^. Endogenous ZNT3 is present at only low level in PC12 cells and transfected ZNT3 protein traffics to synaptic-like microvesicle (SLMV) membranes providing a robust system to assay transporter function. To validate the SLMV enrichment method untransfected PC12 cells were fractionated by differential centrifugation to separate nuclear, endosomal/mitochondrial and SLMV fractions^19^. The three fractions were lysed and equal concentrations of protein from each fraction used, separated by SDS PAGE and Western blotted. Ponceau staining was used to verify the equivalence of protein concentration and transfer before probing with anti-synaptophysin1 and anti-v-glut1 antibodies. Signal was strongest in the SLMV fraction consistent with micro-vesicle enrichment (Fig. 3ai).

In four independent experiments, duplicate flasks of PC12 cells were transiently transfected with either wild-type ZNT3 (WT) or mutant ZNT3 (R298C) both tagged with eGFP (Fig. 3aii). To overcome low transfection efficiency, whole cells expressing eGFP were sorted using FACS. Mean eGFP fluorescence per cell was equal for ZNT3 (WT) and ZNT3 (R298C) suggesting similar translation of both proteins (Fig. 3b). The isolated whole PC12 cells containing eGFP were mechanically lysed and the cell contents fractionated by differential centrifugation to obtain the SLMV enriched fraction. Zn^2+^ concentration was directly measured in the SLMV using mass spectrometry, and showed a significantly lower Zn^2+^ concentration in cells transfected with ZNT3 (R298C) when compared to ZNT3 (WT) transfected cells (Fig. 3c). These results suggest a loss of transporter function.

eGFP-tagging provides an opportunity to measure the amount of ZNT3 protein present in the SLMV fraction using fluorimetry. In the SLMV fraction a significantly lower fluorescence signal was evident for ZNT3 (R298C) compared with ZNT3 (WT) (Fig. 3d). ZNT3 (R298C) fluorescence in the SLMV fraction was equivalent to a similar fraction isolated from untransfected cells suggesting an almost complete loss of trafficking of the mutant protein to the vesicle membrane (Fig. 3d).

### ZnT3 null mice have heightened susceptibility to heat-mediated seizures

**Figure 4:**
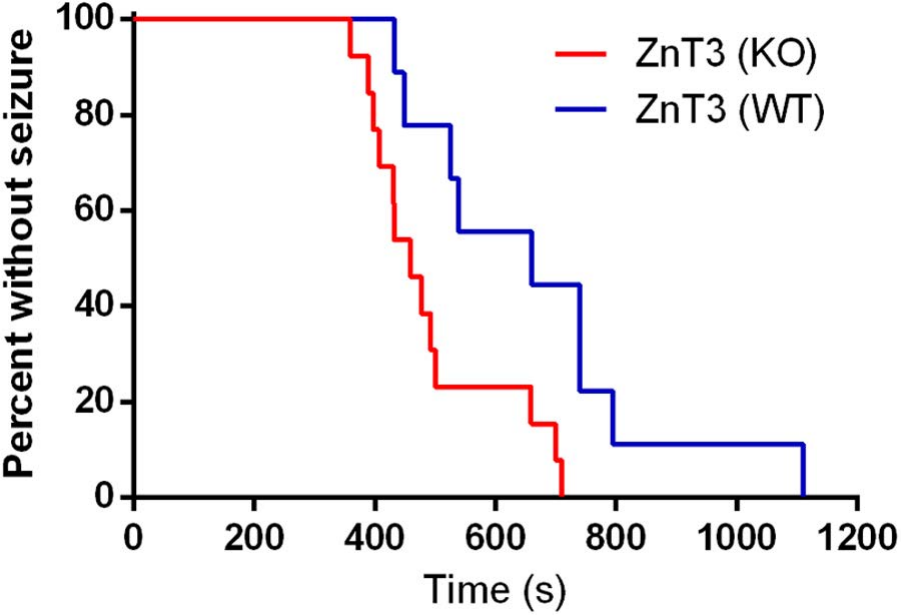
ZnT3 null mice have heightened sensitivity to heat-induced clonic-tonic seizures. Kaplan-Meier curves showing time to first tonic-clonic seizure during exposure to heat for wild-type and ZnT3 knock-out mice homozygous are significantly different (p = 0.01, wild-type, n = 8; ZnT3, n = 11; Mantel-Cox test).

Genetic mouse models of epilepsy based on human FS mutations are more sensitive to heat-induced seizures validating the environmental heating assay^20^,^21^,^22^,^23^. Wild-type and ZnT3 knock-out mice do not differ in gross weight ruling out this confound as a potential basis of differential seizure susceptibility (6.5 ± 0.2 g WT, n = 8 vs. 6.5 ± 0.6 g, KO, n = 11, p > 0.95). Kaplan-Meier survival curve analysis shows that ZnT3 null mice develop heat-mediated clonic-tonic seizure in a significantly shorter time than wild-type mice (Fig. 4). Similarly, average time to clonic-tonic seizure was significantly faster for ZnT3 null mice (694 ± 73 s WT, n = 8 vs. 510 ± 37 s, KO, n = 11, p = 0.03). This is consistent with low synaptic Zn^2+^ levels increasing susceptibility to FS.

## Discussion

We identify the ZNT3 (R298C) variant and show it to be enriched in human FS populations. Although the association does not reach genome wide significance, functional analysis in PC12 cells revealed the variant causes loss-of-function through a trafficking deficit. This loss-of-function is highly likely to predispose to FS as mice null for ZnT3 protein have a heightened sensitivity to thermogenic seizures. Collectively this data strongly implicate *ZNT3* as a susceptibility gene in FS and more generally Zn^2+^ homeostasis as a critical modulator of neuronal excitability.

Genetic factors play an important role in FS susceptibility with about 25% of FS patients having a family history^12^. Furthermore, monozygotic twins have a higher FS concordance than dizygotic twins, strongly implicating a genetic component^11^. The familial epilepsy syndrome most closely associated with FS is genetic epilepsy with febrile seizures plus (GEFS+). FS+ are distinct from FS because they occur outside the usual 6 months to 6 years age range and are often intermixed with afebrile seizures^24^. In families with GEFS + mutations have been identified in sodium channel subunit genes, most commonly *SCN1A* but also *SCN9A* and *SCN1B*, as well as the GABA receptor subunit *GABRG2* and the presynaptic protein syntaxin 1B (*STX1B*)^25^,^26^,^27^,^28^,^29^.

For the more common simple FS, much less of the genetic contribution is known. A genome-wide association study showed a number of common variant associations with FS, all with odds ratios under 2^30^. Two SNPs were replicated in the study, one in the known epilepsy gene *SCN1A* and another in a transmembrane protein of as yet unclear function, *ANO3*. Two further SNPs were associated overall but not replicated, one in *SCN2A* and the other in a non-coding region implicated as a quantitative trait loci for serum Mg^2+^ levels. Furthermore, a quantitative trait locus-based study of sensitivity to environmental-heat induced seizures in mice found an association between seizure susceptibility and *Srp9,* which encodes a cytoplasmic ribonucleoprotein complex^31^. Subsequent analysis in humans showed an association of a *SRP9* promoter region SNP with both FS and temporal lobe epilepsy^31^. Variation in *Fgf13, HCN2,* and *KCC2* have also been implicated in FS^32^,^33^,^34^,^35^. Our findings add to these reports indicating that a variant in *ZNT3* causing loss-of-function acts as a risk allele of moderate effect rather than a SNP of small effect or a dominant Mendelian allele.

The cellular assays confirm that the R298C variant leads to a marked loss of ZNT3 function. Given the equivalent translation of both ZNT3 (WT) and ZNT3 (R298C) proteins in PC12 cells, the lack of expression of ZNT3 (R298C) in the SLMV fraction points to a specific trafficking deficit. ZNT3 forms homodimers which are critical for the correct subcellular localization^18^. Interestingly, Salazar and colleagues showed that reducing dimerization of ZNT3 reduced trafficking to the SLMV fraction. Site-directed mutagenesis isolated the carboxy-terminus as a critical regulator of dimerization^18^. The ZNT3 (R298C) variant falls within the carboxy-terminus and is therefore well placed to disrupt dimerization, although future studies are required to determine the molecular basis of the trafficking deficit.

Multiple potential mechanisms may underlie increased excitability as extracellular Zn^2+^ interacts with a range of ion channels, receptors and transporters^15^,^36^,^37^,^38^. NMDA receptors, however, stand out as a target for Zn^2+^ because of their high sensitivity with levels as low as 10 nM producing significant inhibition under specific conditions^39^. Indeed, activity-dependent release of Zn^2+^ at hippocampal synapses modulates NMDA-mediated excitability^14^. This impact on excitability was limited to higher stimulation frequencies, a situation likely to occur during neuronal hyperexcitability induced by fever^40^. Here we show that ZnT3 knock-out mice are more sensitive to heat-induced seizures, a phenomenon we have previously shown to model a human FS phenotype. Importantly, Vergnano and colleagues demonstrated that trains of evoked NMDA receptor-mediated synaptic events are significantly larger in the ZnT3 knock-out mouse^14^. We propose a model of pathogenesis in which heat-mediated increases in hippocampal neuronal excitability are not constrained in patients harbouring ZNT3 (R298C) due to reduced inhibition of NMDA receptors by Zn^2+^.

Environmental Zn^2+^ deficiency also appears to predispose to FS. Several clinical studies report low blood and CSF Zn^2+^ levels in children who have been diagnosed with FS^7^,^8^,^9^,^10^. Our results provide the first genetic evidence that disruption in Zn^2+^ homeostasis can lead to an increased FS susceptibility. Interestingly, da Rocha and colleagues reported that serum Zn^2+^ levels appear to be influenced by a common *SLC30A3* variant, although the mechanistic basis of this is unclear^41^. This cements the idea that low Zn^2+^ levels confer FS susceptibility through both environmental and genetic means.

## Materials and Methods

### Clinical Evaluation and Patient Sample

All experimental protocols were approved by The Human Research Ethics Committee of Austin Health, Melbourne, Australia, and the Commission for Medical Ethics of the University of Antwerp, Antwerp, Belgium. All methods were carried out in accordance with the approved guidelines of The Human Research Ethics Committee of Austin Health, Melbourne, Australia, and the Commission for Medical Ethics of the University of Antwerp, Antwerp, Belgium. Informed consent was obtained from all human subjects. A comprehensive assessment of each patient was obtained using a validated seizure questionnaire, clinical evaluation by an experienced epileptologist, and review of relevant medical records and clinical investigations. Where possible patients also underwent neurological and general medical examination. Previous clinical records and reports of relevant investigations were obtained if available. We studied 286 unrelated patients of European ancestry recruited from Australia or Belgium diagnosed with FS and 643 control individuals without FS from the same populations. Whole blood was obtained and genomic DNA extracted using a Qiagen QIAamp DNA Maxi Kit (Valencia, CA).

### PCR, Sanger Sequencing and Marker Analysis

The *SLC30A3* gene was amplified using gene-specific primers (oligonucleotides available on request) designed to the full-length reference human gene transcript (NM_003459; NCBI Gene http://www.ncbi.nlm.nih.gov/). Amplification reactions were cycled using a standard protocol on a Veriti Thermal Cycler (Applied Biosystems, Carlsbad, CA) at 60 °C annealing temperature for 1 minute. Bidirectional sequencing of all exons and flanking regions was completed with a BigDye^TM^ v3.1 Terminator Cycle Sequencing Kit (Applied Biosystems), according to the manufacturer’s instructions. Sequencing products and microsatellite markers were resolved using a 3730xl DNA Analyzer (Applied Biosystems). All sequencing chromatograms were compared to published cDNA sequence; nucleotide changes were detected using Codon Code Aligner (Codon Code Corporation, Dedham, MA).

### DNA constructs and mutagenesis

A clone of human *SLC30A3* (ZNT3) CDS (NCB1 XM_006712100) in pUC 57 was obtained from GenScript (Piscataway, NJ, USA). PCR was used to introduce the variant into this construct, and the construct was tagged at the carboxy-terminal with eGFP. Tagged constructs were inserted into a pCDNA 3.1 vector. DNA for transfection was assessed for integrity and quantitated by both Nano-drop 2000 (Thermo Scientific) and gel electrophoresis. OD260/280 ratios were 1.85–1.86. For transfection DNA was diluted to 1 ug/ul in sterile water.

### Cell Culture

Pheochromocytoma (PC12) cells were grown and maintained in Dulbecco’s Modified Eagle Medium (DMEM) supplemented with 10% Fetal bovine serum (FBS), 5% horse serum and 1% Penicillin-Streptomycin and held at 37 ^o^C in 5% CO_2._ Cells were transfected by electroporation (AMAXA Cell Line Nucleofector kit V, Lonza, Basel, Switzerland). After 72 hours they were exposed to 25 μM ZnSO_4_ (in DMEM) and incubated for 1 hour followed by 3 washes in phosphate-buffered saline (PBS). Cells were detached enzymatically (Accutase, Sigma Aldrich, Australia), pelleted, and resuspended in cell-sorter medium (PBS supplemented with 2% FBS and 1.5 mM EDTA). Non-transfected cells were used as a control. A 3 × 10^6^ cells/ml^−1^ suspension was prepared for flow cytometry. Transfected cells were then separated by Fluorescence Activated Cell Sorting (FACS) using a BD Aria 111 cell sorter (BD Biosciences, San Jose, CA, USA).

### Cell fractionation

FACS sorted cells were pelleted and resuspended in intracellular buffer (150 mM NaCl, 10 mM HEPES pH 7.4, 1 mM MgCl_2_, and Complete protease inhibitors (Roche, Basel, Switzerland)) and lysed by sonication for 1 second. Cell lysis was confirmed by microscopy. The lysates were fractionated by differential centrifugation as described previously^19^. Briefly, the lysed cells were centrifuged at 1000 g for 15 minutes to generate a nuclear pellet and the supernatant subsequently centrifuged at 27,000 g for 45 minutes, generating an endosomal/mitochondrial pellet with the final supernatant centrifuged at 200,000 g for 90 minutes to obtain a synaptic–like microvesicle (SLMV) enriched fraction. The SLMV enriched fractions were stored at −80 °C.

### Imaging

Transfected PC12 cells were fixed in 2% paraformaldehyde for 20 minutes and mounted for imaging analysis. Confocal images were obtained using a Zeiss LMS 800 (Zeiss, Jena, Germany) microscope using excitation and emission wavelengths optimised for eGFP.

### Zn^2+^ Analysis

Detergent lysed SLMV’s, from equal numbers of fractionated cells, were digested in concentrated high purity nitric acid (Aristar; BDH, London, UK) overnight at room temperature, and then at 90 °C for 20 minutes. Samples were diluted with 1% nitric acid, and measurements made using a Varian UltraMass inductively coupled plasma mass spectroscopy (ICPMS) instrument (PaloAlto, CA ,USA) under operating conditions suitable for routine multi-element analysis. The instrument was calibrated using blank, 10, 50, and 100 ppb of a certified multi-element ICPMS standard solution (ICP-MS-CAl2-1; AccuStandard) for Mn^2+^, Fe^2+^, Cu^2+^, and Zn^2+^ in 1% nitric acid. Results were obtained from four independent experiments. Mn^2+^ and Fe^2+^ were below levels of detection and Cu^2+^ values were inconsistent across the experimental groups. Zn^2+^ concentrations were untransfected < transfected mutant < transfected wild-type in all experiments. Zn^2+^ in un-transfected cells was deducted from the values in transfected cells prior to analysis. Comparisons between wild-type and mutant were made within experiments, with the wild-type value set as 1.

### Fluorimetry of SLMV fraction

ZNT3 levels in the samples were assayed in duplicate samples by eGFP fluorimetry in a black-walled 384 well plate in the Polarstar Omega Plate Reader (BMG Labtech, Offenburg, Germany). Protocol parameters were: excitation 485–12, emission 520 and gain set at 3077. eGFP protein in lysis buffer was used to generate a standard curve to ensure readings fell in an assayable range.

### Western blotting

Western blotting was carried out according to a previously published method^42^. Protein concentration in samples was assayed by the Bradford method (BioRad, Hercules, CA, USA) and equal protein concentrations from each fraction (80 μg) were separated by electrophoresis on a 12% SDS poly-acrylamide gel, transferred to nitrocellulose by semi-dry Western blotting, and the filter stained with Ponceau S (Sigma, St Louis MO, USA) to confirm successful, even and equivalent protein transfer. Anti-synaptophysin1, 1:3000 (Synaptic Systems, Goettingen, Germany), and anti-vglut1, 1:500 (ab 104899, Abcam, Cambridge UK) primary antibodies and anti-rabbit HRP conjugated 1:3000 (BioRad, Hercules, CA, USA) and anti-sheep/goat HRP conjugated 1:1000 (Silenus, Amrad, Melbourne, Australia) secondary antibodies were used to confirm SLMV enrichment.

### Thermogenic Seizure Testing

All animal experimentation was performed in accordance with ethics approval obtained from the Florey Institute of Neuroscience and Mental Health Animal Ethics Committee. Post-natal 16–17 mice were placed in a container heated to constant 41–42 °C and the time to first tonic-clonic seizure recorded^20^. Mice were sacrificed immediately after the first observed seizure to comply with our animal ethics approval. Comparisons were made between ZNT3 knock-out and their wild-type littermates^43^.

### Statistical analysis

Statistical analysis for association was Fisher’s Exact Test calculated in R (version 2.10, http://R-project.org). Survival curves for each group were analysed using the Mantel-Cox method (GraphPad, CA, USA). Analysis for average time to seizure was completed using an unpaired t-test (GraphPad). Statistical analysis for the functional study used paired t-tests and one way ANOVA as appropriate, see Figure legends (GraphPad). P < 0.05 was set as significant. Graphs are mean ± standard error of the mean.

## Additional Information

**How to cite this article**: Hildebrand, M. S. *et al.* Loss of synaptic Zn^2+^ transporter function increases risk of febrile seizures. *Sci. Rep.* **5**, 17816; doi: 10.1038/srep17816 (2015).

## Supplementary Material

Supplementary Information
