# Supplementary material for: Loss of synaptic Zn2+ transporter function increases risk of febrile seizures
Source: Sci Rep. 2015 Dec 9;5:17816. doi: 10.1038/srep17816 (PMC4673435; doi:10.1038/srep17816)
Supplement: Supplementary Information [file srep17816-s1.pdf]

Supplementary Information

Title: Loss of synaptic Zn^{2+} transporter function increases risk of febrile seizures

Author list: Michael S. Hildebrand, PhD#[1], A. Marie Phillips, PhD#[2,3], Saul A. Mullen, MBBS, PhD#[2], Paul A. Adlard, PhD[2], Katia Hardies, PhD[4,5], John A. Damiano, BSc[1], Verena Wimmer, PhD[2] ,Susannah T. Bellows, BSc[1], Jacinta M. McMahon, BSc[1], Rosemary Burgess, PhD [1], Rik Hendrickx, BSc[4], Sarah Weckhuysen, MD, PhD[4,5], Arvid Suls, PhD[4,5], Peter De Jonghe, MD, PhD[4,5,7], Ingrid E. Scheffer, MBBS, PhD[1,2,6], Steven Petrou, PhD[2], Samuel F. Berkovic, MD[1], Christopher A. Reid, PhD[2 *]

Supplementary Table 1: Results of marker analysis at *SLC30A3* locus using microsatellites

Marker	Region (GRCh38/hg18)	het	Proband 1	Proband 2	Proband 3
D2S2200	11,982,372 - 11,982,774	0.85			
D2S320	18,246,819 - 18,247,033	0.83			
D2S305	19,218,690 - 19,218,962	0.72			
D2S2150	20,332,108 - 20,332,438	0.78			
D2S2144	25,719,533 - 25,719,763	0.84			
D2S174	26,617,005 - 26,617,207	0.8			
D2S2247	27,081,043 - 27,081,196	0.72			
c.892C>T#	27,252,400 - 27,265,200	-			
D2S223	28,115,023 - 28,115,265	0.6			
D2S165	28,380,416 - 28,380,708	0.84			
D2S352	31,278,259 - 31,278,628	0.82			

The small region shared by all three probands and a maximum size of 1.50 Mb is noted in red, additional common markers in orange. #*SLC30A3* variant (ZNT3 R298C)
